# Impact of the COVID-19 pandemic on adults accessing specialist psychiatric care: A cross-sectional Canadian analysis

**DOI:** 10.1371/journal.pone.0346913

**Published:** 2026-04-15

**Authors:** Mary E. Kittur, Brett D. M. Jones, Shayan Imran, Wei Wang, Sheng Chen, Muhammad Ishrat Husain

**Affiliations:** 1 Institute of Medical Science, Temerty Faculty of Medicine, University of Toronto, Toronto, Ontario, Canada; 2 Temerty Centre for Therapeutic Brain Intervention, Centre for Addiction and Mental Health, Toronto, Ontario, Canada; 3 Department of Psychiatry, Temerty Faculty of Medicine, University of Toronto, Toronto, Ontario, Canada; 4 Biostatistics Core, Centre for Addiction and Mental Health, Toronto, Ontario, Canada; 5 College of Public Health, University of South Florida, Tampa, Florida, United States of America; 6 Centre for Mental Health, University Health Network, Toronto, Ontario, Canada; University of the West Indies at Saint Augustine, TRINIDAD AND TOBAGO

## Abstract

**Background:**

The COVID-19 outbreak presented significant psychological challenges, placing individuals with pre-existing psychiatric conditions at disproportionate risk. Identifying the pandemic-related factors driving mental health outcomes in this population is essential to inform targeted interventions during future public health crises.

**Objectives:**

To examine associations between COVID-19-related stressors and psychological symptom severity among Canadian adults with pre-existing mental health conditions during the first two years of the pandemic.

**Methods:**

This cross-sectional study administered a web-based survey to outpatients accessing specialist psychiatric services in Toronto, Canada, between May 2020 and March 2022, aligning with the first five COVID-19 infection “waves” in the region. A series of linear regression models assessed associations between pandemic-related factors (COVID-19 fears, FCV-19S; consumed pandemic-related media; coping responses, Brief-COPE) and symptoms of anxiety (GAD-7) or depression (PHQ-9).

**Results:**

362 participants completed the self-report survey (70% female, mean age 33.8 ± 11.7). Anxiety (60.2%) and depression (59.1%) were the most frequently reported pre-existing psychiatric conditions. Depressive and anxious symptoms met thresholds for “moderate” severity during all five pandemic waves. Greater fear of COVID-19 was associated with increased symptoms of depression (*β* = 0.12, 95% CI: 0.01, 0.22) and anxiety (β = 0.25, 95% CI: 0.16, 0.35). Avoidant and emotion-oriented coping predicted increased psychological symptoms (*p* < .001), while problem-focused coping was associated with lower depression scores (*β* = −0.15, 95% CI: −0.28, −0.02). Frequency of pandemic-related media consumption showed no significant association with any psychological symptoms (*p* > .05).

**Conclusions:**

Canadians with pre-existing mental health conditions experienced persistently elevated psychological symptoms throughout the first two years of the COVID-19 pandemic. COVID-related fear and maladaptive coping emerged as key drivers of symptom severity, while problem-focused coping showed a protective effect, suggesting modifiable treatment targets. Findings underscore the importance of interventions targeting fear attenuation and adaptive coping to protect psychiatric patients during a prolonged public health crisis. Future research should extend investigations globally to determine the broader mental health impact of pandemic stressors in patient populations. Ultimately, findings support the integration of sustained psychological support within emergency response protocols to mitigate the psychological burden of future pandemics and health crises.

## Introduction

The SARS-CoV-2 (COVID-19) outbreak was declared a global pandemic by the World Health Organization (WHO) on March 11, 2020 [1] and is now recognized as the worst public health crisis of the 21^st^ century [2,3]. COVID-19 infections varied from asymptomatic to fatal, with some patients developing severe systemic illnesses such as acute respiratory distress syndrome (ARDS) or acute respiratory failure [4]. As of March 2025, COVID-19 has resulted in an estimated seven million deaths worldwide [5]. The unprecedented scale and transmissibility of the coronavirus quickly overwhelmed healthcare systems [3,6], and most governments responded with strict infection control measures including lockdowns, travel restrictions, and quarantine or social distancing policies [7,8]. While preventative measures were largely successful in slowing disease spread [3,7,9], they did little to address the concurrent mental health crisis that persists to date.

The psychological impact of the pandemic has been well-documented in the general population, with community samples showing increases in depression, stress, fear, anger, and related psychopathology [10–14]. However, COVID-19 posed an even greater threat to people with pre-existing mental health conditions. Virus containment measures are inherently isolating, potentially exacerbating feelings of depression, loneliness, or anxiety [15–17]. Contamination fears and the widespread promotion of infection prevention measures could reinforce existing obsessive-compulsive behaviours and health anxieties [18,19]. Challenges for people with chronic mental health conditions are further compounded by pandemic-related impediments to mental healthcare. Disruptions to essential maintenance treatments, therapeutic laboratory monitoring, and specialized services such as electroconvulsive therapy (ECT) can increase the risk of relapse, self-harm, and suicidal ideation [18,20,21]. Given these heightened vulnerabilities, it is essential to identify and support the mental health needs of psychiatric patients during a global health crisis.

Psychological interventions are a feasible solution to protect mental health during a pandemic, and identifying relevant stressors driving pandemic-emergent psychopathology is essential to clarify treatment targets. A central stressor during the COVID-19 pandemic was fear of the virus itself. The invisibility and transmissibility of infectious diseases can drastically reduce perceptions of personal agency [22], and the global scale of the recent pandemic [23], combined with the extremity of preventative responses [24], suggests that fears of COVID-19 may have been particularly pervasive. COVID-19 was also the first global health crisis to occur in a predominantly digital world [25], amplifying the psychological risks of media content and engagement. For instance, the prominence of COVID-related news [26], the inherently negative content [27], and the spread of virus-related misinformation [28] have all been linked to worsened psychological wellbeing. Pandemic-related stressors may be particularly detrimental for those with existing mental health challenges who may already struggle with adaptive emotion regulation and coping strategies [23,29]. The effects of specific pandemic-related stressors remain understudied in psychiatric populations, and isolating these underlying mechanisms is critical to guide the development of relevant and effective psychological interventions.

The objective of this cross-sectional study was to examine associations between COVID-19 related stressors and psychological symptom severity in a sample of Canadian adults with pre-existing psychiatric conditions during the first two years of the pandemic. We tested three primary hypotheses. First, we hypothesized that specific fears of COVID-19 would predict greater severity of both depressive and anxious symptoms. Second, we predicted that more frequent consumption of COVID-19-related media would be associated with increases in depression and anxiety. Finally, our exploratory hypothesis proposed that coping strategies used by individuals with mental health conditions would moderate the severity of psychological symptoms. Taken together, these insights may inform the development of targeted psychological interventions to ensure that the specific needs of psychiatric patients are adequately addressed during future pandemics and health crises.

## Materials and methods

### Study design

This was a cross-sectional observational study conducted and reported in line with STROBE guidelines for cross-sectional research [30]. We developed a web-based survey containing a series of self-report assessments and questionnaires deemed suitable for online use. Given the time-sensitive nature of the COVID-19 outbreak, convenience sampling was used to recruit study participants from two adult outpatient clinics at the Centre for Addiction and Mental Health (CAMH), a tertiary care psychiatric hospital in Toronto, Canada. All individuals presenting at these clinics for psychiatric consultation or follow-up between May 2020 and March 2022 were offered the opportunity to participate. Opting in to receive the one-time online survey was entirely voluntary and there were no incentives for participation.

### Study sample

The first participant was recruited on 19 May, 2020 and the final enrolment occurred on 26 March, 2022. This two-year assessment period aligned with the first five COVID-19 infection waves in Ontario, Canada (May-Aug 2020, Sep 2020-Feb 2021, Mar-Aug 2021, Sep-Nov 2021, and Dec 2021-Mar 2022). Pandemic waves were defined as periods of infection surges followed by declines, according to regional public health guidelines (i.e., Health Canada positive COVID-19 case data for Ontario [31]).

#### Inclusion criteria.

Participant eligibility criteria were kept intentionally broad in order to capture the full spectrum of patient experiences. In order to participate, subjects were required to be at least 18 years of age, able to read and write in English, and able to complete online consent and survey procedures using the study web platform.

#### Exclusion criteria.

There were no exclusion criteria for participation.

### Study procedures

All study activities were conducted remotely to accommodate local pandemic-related restrictions to face-to-face research. All participants provided written informed consent prior to initiation of the web-based survey. Patients accessing care at the two outpatient clinics were informed about the study by clinic administrators, who followed an approved telephone script emphasizing that study participation was voluntary and independent from clinical care. Verbally consenting individuals were emailed a link to the anonymized survey, hosted on the secure web-based Research Electronic Data Capture platform (REDCap [32,33]) at CAMH. The REDCap link directed subjects to an electronic version of the REB-approved study consent form which did not proceed to the study survey until they had reviewed and agreed to consent terms via electronic signature. Participants were advised that they could skip questions they did not wish to answer and could withdraw from the study at any time without providing a reason. The survey incorporated automatic safety checks to notify research staff of potential safety concerns within questionnaire responses. In the event of a safety flag, subjects received a notification with standardized safety directions and any necessary follow-up was facilitated by the lead investigator.

### Ethical considerations

All study procedures, telephone scripts, and participant-facing materials were reviewed and approved by the local Research Ethics Board at CAMH (REB identifier: 057/2020). All participants provided written informed consent prior to initiation of study procedures. The REB-approved consent form outlined study procedures, privacy and data use parameters, and research team contact information given the remote format. The consent form affirmed that no personally identifiable information would be collected or stored, and that the purpose of the study was to aggregate anonymized responses to identify general mental health needs of people with mental health conditions during the pandemic. Participants were not offered any incentives for participation. They were also reminded that participation was voluntary and separate from clinical care, and that they could skip questions or withdraw entirely at any point without providing a reason.

### Measures

The web-based survey consisted of eight self-report questionnaires assessing demographics, COVID-19 related factors, and psychological outcomes. Three measures pertaining to perceived disability, quality of life, and resilience were not relevant to the present study objectives and are not reported herein. These results have not yet been published but will be used in future analysis relating to the COVID-19 experiences of psychiatric patients.

#### Clinical outcomes.

Primary clinical outcomes were self-reported symptoms of depression and anxiety. Depression was measured using the 9-item Patient Health Questionnaire Scale (PHQ-9 [34]), a validated self-report assessment corresponding to the nine Diagnostic and Statistical Manual of Mental Disorders (DSM-5) criteria for major depressive disorder [35]. Symptom frequency is rated over the past two weeks on a four-point Likert scale ranging 0 (“not at all”) to 3 (“nearly every day”), where higher scores indicate greater depression severity. Total scores range 0–27, with scores of 5, 10, 15, and 20 defined as mild, moderate, moderately-severe, and severe depression, respectively [34]. Anxiety was assessed using the 7-item Generalized Anxiety Disorder Scale (GAD-7 [36]), a self-report scale measuring seven symptoms of DSM-5 anxiety disorders. Respondents rate symptom frequency over the preceding two weeks on a four-point scale ranging 0 (“not at all”) to 3 (“nearly every day”). Total scores range 0–21, with higher score indicating greater anxiety. Scores of 5, 10, and 15 represent mild, moderate, and severe anxiety, respectively [36].

#### COVID-19 stressors.

Primary COVID-related predictors in the present study included specific fears of COVID-19 and pandemic-related media consumption. COVID-19 fears were assessed using the 7-item Fear of COVID-19 Scale (FCV-19S [37]), a measure developed specifically for the COVID-19 pandemic which has shown strong psychometric properties and construct validity across samples and languages [38]. Subjects indicate their agreement with seven fear responses applied to the context of COVID-19 (e.g., “I am most afraid of coronavirus-19”, “My hands become clammy when I think about coronavirus-19”). Responses span a five-point Likert scale from 1 (“Strongly disagree”) to 5 (“strongly agree”), with total score ranging 7–35 and higher scores indicating greater fear of COVID-19. Pandemic-related media use was assessed using four self-report items. Subjects were asked to select their primary sources of COVID-19-related news from a list of media platforms including Health Canada, WHO, television, social media sites, and “other”. Participants indicated average time per day spent reading or listening to COVID-19 related news on a categorical scale ranging “no time at all” to “6+ hours”, as well as any change in media use frequency since pandemic onset (i.e., increase, decrease, or unchanged). If applicable, subjects further quantified media consumption increase on a categorical scale ranging “30mins to 1 hour more than usual” to “over 4 hours more than usual”.

#### Exploratory measures.

Self-efficacy and coping with COVID-19 were assessed via the Brief Coping Orientation to Problems Experienced inventory (Brief-COPE [39]), a 28-item scale assessing coping responses to an administrator-specified stressful event (i.e., COVID-19 in the present study). Responses correspond to three domain subscales of problem-focused, emotion-focused, and avoidant coping strategies. These subscales align with the original COPE scale [40] and have demonstrated content validity in previous clinical trials [41,42]. Eight brief-COPE items pertain to problem-focused coping (e.g., “I've been concentrating my efforts on doing something about the situation I'm in”), 12 items refer to emotion-focused coping (e.g., “I’ve been criticizing myself”), and eight relate to avoidant coping (e.g. I’ve been using alcohol or other drugs to help me get through it”). All items are rated on a four-point Likert scale ranging from 1 (“I haven’t been doing this at all”) to 4 (“I’ve been doing this a lot”), where higher scores indicate greater use of the respective coping strategy.

### Statistical analysis

Apriori power analysis estimated that a total sample of 200 participants was needed to detect bivariate associations of 0.25 or above between pairs of predictor and outcome variables. Multivariate analyses had smaller minimum detectable effects given that the addition of covariates reduces model standard error. Power calculations assumed 80% power and a large proportion of missing entries (~40%). All statistical analyses were performed in R (version 4.2 [43]) assuming a significance level of α = 0.05. A series of independent linear regression models were constructed; first, simple linear regression assessed bivariate relationships between pairs of predictor and outcome variables, then multiple regression tested the strength of these linear relationships after adjusting for sociodemographic covariates. Predetermined demographic covariates included gender, age, marital status, education, self-reported psychiatric diagnosis, and COVID-19 wave. Though we initially planned Full Information Maximum Likelihood (FIML) estimation to account for missing data, review of the final dataset indicated that imputation would be ineffective as incomplete entries left the majority of COVID-related questions blank. It was therefore determined that only questionnaire completers would be included in the final sample. Given the exploratory nature of study hypotheses, we did not adjust regression analyses for multiple comparisons [44].

## Results

### Sample characteristics

A total of 362 subjects completed the survey and were included in the final analyzed cohort. 479 individuals accessed the survey and provided at least one response, yielding a completion rate of 75.6%. Descriptive characteristics of the 362 participants are presented in Table 1. Over two-thirds of this sample were cisgender female (70.4%), with an average age of 33.8 years (SD = 11.7). Most subjects had completed some degree of post-secondary education (78.2%) and approximately one-third were employed full-time (32.0%). More than three-quarters of the sample (77.3%) reported a pre-existing psychiatric condition diagnosed prior to the current clinic interaction. The most prevalent pre-existing diagnoses were anxiety (60.2%) and depression (59.1%). The majority of respondents (85.4%) reported experiencing new or worsening psychiatric symptoms since the pandemic began.

**Table 1:** Descriptive characteristics of the 362 study participants.

	N (%)	COVID-19 wave^a^				
		Wave 1: May-2020	Wave 2: Sep-2020	Wave 3: Mar-2021	Wave 4: Sep-2021	Wave 5 + : Dec-2021
Total N	362 (100%)	119	72	98	43	30
Age, years- mean (SD)	33.8 (11.7)	35.6 (11.4)	32.4 (11.6)	33.6 (11.9)	32.0 (12.6)	32.7 (11.1)
*Gender*						
**Female**	**255 (70.4%)**	87	52	63	30	23
Male	87 (24.0%)	28	17	25	12	5
Other^b^	20 (5.5%)	4	3	10	1	2
*Education level*						
<Grade 12	16 (4.4%)	3	2	8	2	1
High school	63 (17.4%)	18	14	16	9	6
**Any post-secondary**	**283 (78.2%)**	98	56	74	32	23
College	102 (28.2%)	27	22	30	13	10
Undergraduate	106 (29.3%)	39	22	25	15	5
Graduate	75 (20.7%)	32	12	19	4	8
*Employment status*						
**Full-time**	**116 (32.0%)**	36	25	28	11	16
Part-time	31 (8.6%)	8	5	10	5	3
Student	43 (11.9%)	11	13	7	8	4
Sick leave	58 (16.0%)	26	6	17	7	2
Retired	5 (1.4%)	1	1	2	1	0
Unemployed	65 (18.0%)	18	14	24	5	4
Part-time due to COVID-19	8 (2.2%)	1	2	3	2	0
Laid off due to COVID-19	29 (8.0%)	17	6	5	1	0
No response	7 (1.9%)	1	0	2	3	1
***Psychiatric diagnosis = yes***	**280 (77.3%)**	104	60	70	26	20
**Depression**	**214 (59.1%)**	71	46	58	24	15
**Anxiety**	**218 (60.2%)**	72	52	56	22	16
Bipolar	9 (2.5%)	26	8	11	0	4
Schizophrenia	1 (0.3%)	1	0	0	0	0
Substance use	16 (4.4%)	7	4	3	0	2
Other	70 (19.3%)	21	14	24	5	6
***New or worsening symptoms = yes***	**309 (85.4%)**	100	60	86	35	28
Few/mild	92 (25.4%)	38	14	22	9	9
**Several/moderate**	**113 (31.2%)**	35	20	32	13	13
Many/severe	104 (28.7%)	27	26	32	13	6

^a^ Defined according to Health Canada COVID-19 positive case data for Ontario [31].

^b^ n = 4 non-binary, n = 3 gender-fluid, n = 8 transgender, n = 5 undisclosed.

### Clinical and pandemic-related outcomes

Table 2 summarizes outcomes on clinical and pandemic-related measures both overall and by successive COVID-19 wave. Overall mean PHQ-9 score was 16.5 (SD = 6.4), corresponding to “moderately severe” depression [34]. PHQ-9 scores remained in the “moderately severe” threshold during all pandemic waves except wave five and beyond, when symptoms decreased to “moderate” severity (mean 13.3, SD = 5.7). Overall GAD-7 score was 13.3 (SD = 5.7), representing “moderate” anxiety [36]; GAD-7 severity remained in the “moderate” category during all five waves. Both depression and anxiety peaked during wave three (Mar-Aug 2021).

**Table 2:** Clinical outcomes and COVID-19 media use behaviours.

	Total (mean, SD)	COVID-19 wave^a^				
		Wave 1: May-2020	Wave 2: Sep-2020	Wave 3: Mar-2021	Wave 4: Sep-2021	Wave 5 + : Dec-2021
PHQ-9	16.5 (6.4)	15.3 (7.0)	17.0 (6.0)	**18.4 (5.8)**	17.0 (5.8)	13.3 (5.7)
GAD-7	13.3 (5.7)	12.4 (6.0)	13.3 (5.2)	**14.4 (5.5)**	14.0 (5.8)	12.5 (5.5)
FCV-19S	16.5 (6.4)	17.0 (6.2)	17.1 (6.7)	15.5 (6.9)	15.7 (6.8)	18.1 (3.9)
*Brief-COPE*						
Problem-focused	18.5 (5.3)	18.1 (5.2)	18.6 (5.3)	19.1 (5.7)	17.4 (4.7)	19.3 (4.5)
Emotion-focused	27.7 (5.4)	27.1 (5.6)	27.4 (5.5)	28.9 (5.4)	27.3 (5.0)	27.7 (3.6)
Avoidant	15.8 (4.3)	15.3 (4.1)	15.7 (4.1)	16.7 (4.3)	15.1 (4.5)	15.8 (4.7)
*Daily COVID-19 media consumed- n (%)*						
No time	57 (15.7%)	13	8	20	11	5
**0-2 hours**	**269 (74.3%)**	89	54	71	30	25
2-4 hours	29 (8.0%)	12	9	6	2	0
4-6 hours	4 (1.1%)	2	1	1	0	0
6 + hours	2 (0.6%)	2	0	0	0	0
N/A	1 (0.3%)	1	0	0	0	0
*Media use change- n (%)*						
**Increased**	**211 (58.3%)**	81	47	51	19	13
Decreased	38 (10.5%)	13	4	14	7	0
Unchanged	112 (30.9%)	24	21	33	17	17
N/A	1 (0.3%)	1	0	0	0	0

^a^Defined according to Health Canada COVID-19 positive case data for Ontario [31].

Brief-COPE: Brief Coping Orientation to Problems Experienced inventory, FCV-19S: Fear of COVID-19 scale, GAD-7: Generalized Anxiety Scale, PHQ-9: Patient Health Questionnaire.

#### Specific fears of COVID-19.

Mean score on the Fear of COVID-19 scale (FCV-19S) was 16.5 (SD = 6.4; Table 2). FCV-19S scores peaked during wave five and beyond (18.1, SD = 3.9) and were lowest during wave three (15.5, SD = 6.9). Table 3 presents results of simple linear regression between FCV-19S and psychological outcomes. Though neither PHQ-9 or GAD-7 measures were normally distributed, linear regression was still deemed appropriate as the normality of residual distributions remained acceptable in all regression analyses. Bivariate linear regression revealed a significant positive association between both FCV-19S and PHQ-9 (*β* = 0.12, 95% CI: 0.01, 0.22, *p* = 0.026 Table 3) and between FCV-19S and GAD-7 (*β* = 0.25, 95% CI: 0.16, 0.35, *p* < .001; Table 3).

**Table 3:** Bivariate linear regression examining fear of COVID-19 as a predictor of depression or anxiety.

Predictor	Model 1: PHQ-9			Model 2: GAD-7		
	*β* (SE)	95% CI	*p*-value	*β* (SE)	95% CI	*p*-value
FCV-19S	0.12 (0.05)	0.01, 0.22	**0.026**	0.25 (0.05)	0.16, 0.346	**<.001**
F	4.00			30.12		
R^2^	**0.01**			**0.08**		

*β*: standardized beta coefficient, CI: confidence interval, FCV-19S: Fear of COVID-19 scale, GAD-7: Generalized Anxiety Scale, PHQ-9: Patient Health Questionnaire, SE: standard error.

Results of multiple regression analyses are presented in supplementary materials (see S1 Table). After adjusting for gender, age, marital status, education level, self-reported psychiatric diagnosis, and COVID-19 wave, the direct effect of FCV-19S on PHQ-9 was rendered marginally significant (*β* = 0.098, 95% CI [−0.01, 0.20], *p* = 0.068; S1 Table). Independent predictors in the PHQ-9 model included subject education level (*p* < .001), driven by a significant negative association between graduate education and PHQ-9 (*β* = −4.78, 95% CI: −8.27, −1.29, *p* = 0.008; S1 Table), and COVID-19 wave, driven by a significant positive association with wave three (*β* = 3.01 95% CI: 1.27, 4.75, *p* < .001; S1 Table). Multiple regression on anxiety retained a strong positive association between FCV-19S and GAD-7 even after adjusting for covariates (*β* = 0.23, 95% CI: 0.14, 0.34, *p* < .001; S1 Table). No additional predictors emerged in the GAD-7 model.

#### Frequency of consumed COVID-19 media.

Outcomes on COVID-19 media-related variables are presented in Table 2. More than half of participants (58.3%) reported an increase in media use since pandemic onset. The most commonly utilized media sources were Health Canada (54.4%) and television (49.4%), with social media sites the least frequently endorsed (10.8–19.1%). Responses on the primary predictor of COVID-19 media consumption were significantly imbalanced: the majority of participants (74.3%) endorsed the same category of “0-2 hours per day” of media use, and less than 2% reported the upper extremes of “4-6 hours” (n = 4) or “6+ hours” (n = 2). These few extreme responses contributed most of the variance in linear models, and the results of analyses are therefore interpreted with caution.

Generalized linear regression assessing associations between COVID-19 media frequency and clinical outcomes is summarized in Table 4. Linear regression on PHQ-9 indicated no significant effect of pandemic-related media use (*p* > .05). In the GAD-7 model, simple regression initially suggested a negative association between media consumption and anxiety symptoms (*p* = 0.004). However, this effect was primarily driven by the few extreme “6+ hours” responses (*β* = −13.7, 95% CI: 21.6, −5.82, *p* < .001; Table 4), and the association became non-significant (*p* > .05) after omitting the outlying category in post-hoc sensitivity analyses.

**Table 4:** Generalized linear regression examining COVID-19 media use as a predictor of depressive or anxious symptoms.

Predictor	PHQ-9 model				GAD-7 model			
	*β* (SE)	95% CI	*p*-value	χ^2^	*β* (SE)	95% CI	*p*-value	χ^2^
*Daily COVID-19 media consumed*	Ref.		0.056	9.21	Ref.		**0.004**	**15.12**
0-2 hours	−1.52 (0.95)	−3.39, 0.35	0.11		−0.59 (0.82)	−2.19, 1.02	0.48	
2-4 hours	−1.11 (1.50)	−4.05, 1.83	0.46		1.11 (1.33)	−1.50, 3.72	0.40	
4-6 hours	−0.60 (3.30)	−7.07, 5.87	0.86		2.80 (2.89)	−2.86, 8.46	0.33	
6 + hours	**−12.85 (4.59)**	**−21.8, −3.85**	**0.005**		**−13.7 (4.02)**	**−21.6, −5.82**	**<.001**	
AIC	2296.2				2242.2			
Residual deviance	13993				10958			

AIC: Akaike information criterion, *β:* standardized beta coefficient, CI: confidence interval, FCV-19S: Fear of COVID-19 scale, GAD-7: Generalized Anxiety Scale, PHQ-9: Patient Health Questionnaire, Ref.: reference level, SE: standard error.

#### Coping strategies and psychological symptoms.

**Fig 1:**
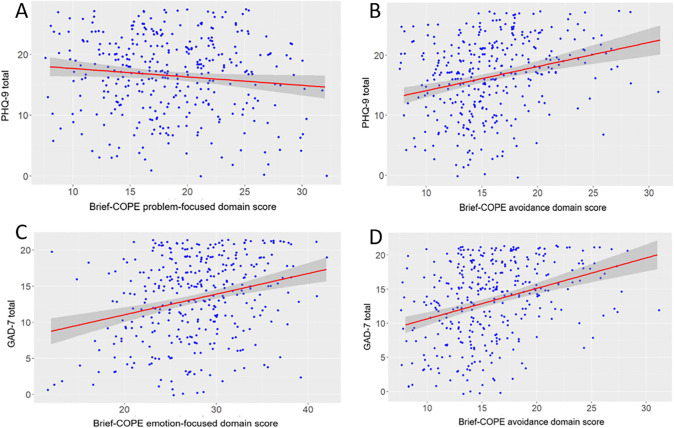
Significant outcomes from bivariate linear regression of Brief-COPE subscales on psychological outcomes. A. Problem-focused coping on PHQ-9. B. Avoidant coping on PHQ-9. C. Emotion-focused coping on GAD-7. D. Avoidant coping on GAD-7. All associations statistically significant at *p* < .05..

Outcomes on Brief-COPE emotion-focused, avoidant, and problem-focused subscales are presented in Table 2. Each domain subscale was independently regressed on psychological symptoms in a series of six bivariate models. Statistically significant associations are presented in Fig 1 below, with full analyses reported in S2 Table. Simple regression on PHQ-9 revealed a significant negative association with problem-focused coping (*β* = −0.14, 95% CI: −0.27, −0.01, *p* = 0.04; Fig 1), a significant positive association with avoidant coping (*β* = 0.40, 95% CI: 0.25, 0.56, *p* < .001; Fig 1), but no association between emotion-focused coping and PHQ-9 (*p* > .05; S2 Table). Bivariate regression on GAD-7 revealed significant positive associations with emotion-focused coping (*β* = 0.29, 95% CI: 0.18, 0.39, *p* < .001; Fig 1) and avoidant coping (*β* = 0.45, 95% CI: 0.31, 0.58, *p* < .001; Fig 1), but no significant association with problem-focused coping (*p* > .05; S2 Table).

Multiple regression analyses further examined associations between Brief-COPE subscales and psychological outcomes after adjusting for sociodemographic covariates (see supplementary materials S3–S6 Tables). Multiple regression on PHQ-9 (S3 and S4 Tables) retained a significant negative association with problem-focused coping (*β* = −0.15, 95% CI: −0.28, −0.02, *p* = 0.025; S3 Table) and a significant positive association with avoidant coping (*β* = 0.28, 95% CI: 0.12, 0.44, *p* < .001; S4 Table). Multiple regression on GAD-7 (S5 and S6 Tables) showed that both emotion-focused (*β* = 0.24, 95% CI: 0.13, 0.35, *p* < .001; S5 Table) and avoidant coping (*β* = 0.34, 95% CI: 0.20, 0.48, *p* < .001; S6 Table) remained significantly and positively associated with GAD-7. Multiple regression on PHQ-9 identified COVID-19 wave and subject education as independent predictors of PHQ-9 (*p* < .01; S3 and S4 Tables). Subject education level emerged as an independent predictor of GAD-7 in the emotion-focused model (*p* = 0.028; S5 Table), but not the avoidant coping model (*p* < .001; S6 Table).

## Discussion

This cross-sectional study examined associations between COVID-19 related stressors and psychological symptom severity in a sample of Canadian adults with pre-existing psychiatric conditions during the first two years of the pandemic. Participants reported persistently elevated psychological symptoms throughout the two-year period, with the majority reporting symptom worsening or relapse relative to pre-pandemic. Fear of COVID-19 was associated with significant increases in depression and anxiety, and both avoidant and emotion-focused coping predicted greater psychological symptoms. Conversely, problem-focused coping showed a possible protective effect in predicting lower depression severity. Although many participants reported increased media use since the pandemic began, frequency of consumed COVID-19-related media was not significantly associated with psychological outcomes. Taken together, study findings suggest sustained psychiatric burden in a psychiatric patient population and highlight key pandemic-related mechanisms driving psychological outcomes.

This two-year cross-sectional study represents one of the longest continuous assessments of psychological stressors in people with existing mental health conditions during COVID-19, facilitating insights into both chronic symptom trends and acute fluctuations. While previous studies have also demonstrated the psychological burden of COVID-19 in psychiatric populations [45–47], most are limited to the first few months of the pandemic, leading to suggestions that any such effects were transient [48–50]. Our findings reveal sustained psychological symptoms up to two years after the initial outbreak, underscoring the need for both acute and long-term psychological supports during a chronic public health crisis. Notably, we identified a peak in depression severity during wave three, coinciding with the first public rollout of COVID-19 vaccination in Ontario, Canada [51]. This may suggest a critical period for therapeutic intervention, as individuals with psychiatric conditions may experience heightened distress as result of perceived barriers to vaccination access [52] or susceptibility to vaccine misinformation [53].

Beyond prevalence trends, this cross-sectional analysis also reveals key factors driving pandemic-emergent psychopathology in psychiatric patients. Previous research has similarly identified fear of COVID-19 as a psychological risk factor in both general [48,54–56] and psychiatric populations [46,47,57,58]. The present work found that fear remained a robust predictor of anxiety even after controlling for demographic variability, highlighting fear attenuation as a core therapeutic target. On the other hand, we did not observe an association between consumed COVID-19-related media and psychological symptoms, failing to replicate a previously reported dose-response relationship between pandemic-related media exposure and heightened psychopathology [59–61] and contrasting suggestions of a COVID-19 “infodemic” impacting populations with [26,62,63] and without [13,64–66] mental health conditions. However, media-related conclusions in this work are limited by our imbalanced response distribution, as the majority of study participants reported the same 0–2 hours/day of pandemic-related media consumption in the context of broad two-hour measurement intervals. Limitations notwithstanding, most of our subjects remained below the 2.5 hours/day threshold for negative psychological effects of COVID-19 media [48], and most media exposure in this study came from traditional or government-maintained sources which have been shown to confer minimal psychiatric risk [48,59,67]. While we cannot conclusively interpret our null finding as the result of methodological error versus a true protective effect, findings emphasize the need for more precise and platform-specific evaluations in future research.

Coping behaviours are another key determinant of mental health during a public health crisis [68,69]. In the present study, avoidant coping with COVID-19 showed a broad detrimental impact on both depressive and anxious symptoms, replicating previous findings in psychiatric [61,70] and community cohorts [61,71,72], as well as global patterns suggesting a universal maladaptive effect of avoidant coping with the pandemic [73]. Emotion-focused coping was uniquely associated with heightened anxiety, reflecting nuanced utility of these strategies in the context of COVID-19 [71,72,74]; for instance, the often adaptive strategy of emotional support-seeking became newly dysfunctional during COVID-related restrictions [72,75]. Interestingly, we also replicated a symptom-specific protective effect of problem-focused coping in lowering depression severity [71,76,77], potentially reflecting the particular efficacy of problem-oriented strategies for mitigating feelings of hopelessness and insecurity more often tied to depression. Altogether, this nuanced effectiveness of coping strategies underscores the importance of personalized coping interventions to effectively mitigate maladaptive responses.

It is critical to interpret our Canadian findings within the larger global and epidemiological context. While our cross-sectional analysis is consistent with elevated psychopathology shown in studies of African, Asian, and South American psychiatric patients during COVID-19 [78–80], evidence also suggests that this psychological burden may have been heightened relative to high-income countries [81,82]. Future research should further investigate the generalizability of pandemic stressors across global patient outcomes. One multi-national study found that pandemic-emergent psychological symptoms in low- and middle-income countries (LMICs) persisted for more than a year after the initial outbreak [83], mirroring the sustained symptom trajectory seen in our two-year study. Research in LMICs suggests that COVID-related stressors were magnified by existing structural, economic, and healthcare disparities [84–86], emphasizing the need for cultural comparison in future studies. Nevertheless, COVID-19 fears and maladaptive coping emerge as common psychological stressors across high- and low-income jurisdictions [80,87–89], suggesting that these stressors highlighted in our work may have fundamental cross-cultural relevance. While certain pandemic-related factors may generalize globally, differences in structural and physical compounding factors must be further investigated to inform truly tailored interventions during a long-term health crisis.

### Implications and recommendations

This cross-sectional study highlights the sustained psychological impact of COVID-19 on individuals with pre-existing mental health conditions, identifying pandemic-related fear and maladaptive coping as modifiable underlying mechanisms. These findings support the development of tailored interventions targeting fear attenuation, adaptive coping, and resilience building. through These factors could be addressed by repurposing existing evidence-based strategies such as peer support, cognitive behavioural, self-help, or mindfulness therapies [90]. Incorporating virus-specific psychoeducation may be particularly useful in mitigating problematic fear [66], and media literacy education around public health messaging could encourage healthy engagement with pandemic-related media. Mental healthcare must be a central component of future pandemic preparedness and response, and our findings suggest that practitioners and policymakers prioritize early identification of high-risk individuals, proactive intervention, and pathways for acute and long-term psychological support. Cultural adaptation is also imperative to ensure that intervention strategies do not further amplify structural inequities; for instance, while telepsychiatry effectively bridged clinical care gaps in many high-income countries [91], the digital divide in low and middle-income settings suggests community-based models, as more feasible alternatives. In all, our findings emphasize the importance of tailored, integrated, and equitable psychological interventions to mitigate the impact of public health crises in psychologically-vulnerable populations.

### Limitations and strengths

We acknowledge several limitations reducing the generalizability of study findings. Convenience sampling from a single outpatient psychiatric clinic may have biased our sample toward a higher-functioning and more resourced patient population, limiting generalizability across patient subgroups. For instance, the exclusion of psychiatric inpatients, non-treatment-seeking individuals, and those lacking the necessary resources to for online participation introduces selection bias that further limits generalizability. Given that the experience and consequences of COVID-19 differed across countries and cultures, the present work may have limited applicability outside of an urban Canadian context. Future research must extend patient-focused investigations to compare and contrast pandemic-emergent psychological stressors in patient groups globally. As acknowledged, COVID-19 media use outcomes were highly skewed in the present work, limiting media-related conclusions. Methodologically, our cross-sectional design precludes causal inference regarding pandemic-related effects on psychological outcomes, and does not account for cumulative effects of these stressors over the two-year period. Where possible, future studies should adopt longitudinal designs to accurately capture dynamic pandemic-emergent sequelae. The use of self-report measures in this study may risk subject reporting bias, particularly in the self-disclosure of subjects’ psychiatric diagnoses. Subsequent studies should emphasize more objective clinical measures, though we note that our participants had all presented at specialist psychiatric clinics, suggesting some external verification of their psychiatric status. Finally, we overlooked a potentially critical confound by omitting assessment of long COVID-19. Defined as COVID-related sequelae persisting three or more months after infection [92], long COVID-19 syndrome is increasingly implicated in pandemic-emergent psychopathology [93], and is known to disproportionately affect individuals with existing psychiatric conditions [94]. As such, it is critical that future research determines how the onset and progression of long COVID-19 intersects with symptom trajectories in psychiatric populations.

Our study also demonstrates several strengths. The final sample size (n = 362) exceeded initial power estimates, ensuring sufficient statistical power to detect true associations between pandemic-related stressors and psychological outcomes. Our broad subject eligibility criteria yielded a naturalistic outpatient sample that enhances the validity and real-world applicability of findings. By continuously recruiting across a two-year period (May 2020-March 2022), we captured both acute and chronic pandemic-emergent trends in our patient population. The study is also unique in differentiating outcomes by distinct COVID-19 waves, facilitating insight into critical COVID-19 developments such as virus mutations, vaccine availability, and varying intensity of preventative measures. Finally, our independent assessment of depression and anxiety revealed symptom-specific relevance of pandemic-related factors, further accelerating the development and implementation of personalized interventions. Ultimately, this cross-sectional analysis provides insight into the specific needs of an often overlooked population during COVID-19, identifying modifiable risk factors to guide future pandemic preparedness.

## Conclusion

The COVID-19 pandemic and associated stressors had a profound impact on global mental health, placing individuals with pre-existing psychiatric conditions at disproportionate risk. In this cross-sectional study of Canadian psychiatric patients, we reveal persistently elevated psychological symptoms throughout the first two years of the pandemic. Fear of COVID-19 and maladaptive coping emerged as key mechanisms driving symptom severity, while problem-focused coping showed a potential protective effect, suggesting modifiable treatment targets. These findings underscore the need for interventions targeting fear attenuation, adaptive coping, and resilience building to effectively support psychiatric patients during a global health crisis. Future research should extend these analyses globally to determine the broader psychological impact of across patient groups and further inform culturally-relevant interventions. It is imperative that practitioners and policymakers leverage insights from COVID-19 and integrate relevant and effective psychological interventions into future pandemic response protocols. Ultimately, findings emphasize the importance of proactive, targeted, and sustained psychological support to mitigate the psychological burden of future long-term health crises in people with existing psychiatric vulnerabilities.

## Supporting information

S1 Table Multiple linear regression of COVID-19 fear and sociodemographic covariates on psychological outcomes. (DOCX)

S2 Table Bivariate linear regression analyses of Brief-COPE domain subscales on psychological outcomes. (DOCX)

S3 Table Multiple linear regression of problem-focused coping, COVID-19 fear, and sociodemographic factors on depressive symptoms. (DOCX)

S4 Table Multiple linear regression of avoidant coping, COVID-19 fear, and sociodemographic factors on depressive symptoms. (DOCX)

S5 Table Multiple linear regression of emotion-focused coping, COVID-19 fear, and sociodemographic factors on anxiety symptoms. (DOCX)

S6 Table Multiple linear regression of avoidant coping, COVID-19 fear, and sociodemographic factors on anxiety symptoms. (DOCX)
