# Supplementary material for: Impact of the COVID-19 pandemic on adults accessing specialist psychiatric care: A cross-sectional Canadian analysis
Source: PLoS One. 2026 Apr 15;21(4):e0346913. doi: 10.1371/journal.pone.0346913 (PMC13082661; doi:10.1371/journal.pone.0346913)
Supplement: S1 Table — (DOCX) [file pone.0346913.s001.docx]

**Supplementary Material 1**

**S1 Table.** Multiple linear regression of COVID-19 fear and sociodemographic factors on psychological outcomes

| Predictor | Model 1: PHQ-9 | | | | | Model 2: GAD-7 | | | |
| --- | --- | --- | --- | --- | --- | --- | --- | --- | --- |
|  | *β* (SE) | 95% CI | *p*-value | **χ^2^** | *β* (SE) | | 95% CI | *p*-value | **χ^2^** |
| FCV-19S | 0.10 (0.05) | -0.01, 0.20 | 0.07 |  | **0.23 (0.05)** | | **0.14, 0.34** | **<.001** |  |
| Age | 0.003 (0.04) | -0.07, 0.08 | 0.94 |  | -0.03 (0.03) | | -0.09, 0.34 | 0.38 |  |
| *COVID-19 wave* |  |  | **<.001** | **19.2** |  | |  | 0.09 | 8.18 |
| Wave 1 | Ref. |  |  |  |  | |  |  |  |
| Wave 2 | 1.45 (0.94) | -0.39, 3.29 | 0.13 |  | 0.36 (0.83) | | -1.27, 1.99 | 0.66 |  |
| Wave 3 | **3.01 (0.89)** | **1.27, 4.75** | **<.001** |  | 1.79 (0.78) | | 0.27, 3.31 | 0.02 |  |
| Wave 4 | 1.45 (1.16) | -0.82, 3.72 | 0.21 |  | 1.35 (1.02) | | -0.65, 3.35 | 0.18 |  |
| Wave 5+ | -1.98 (1.33) | -4.59, 0.63 | 0.14 |  | -0.74 (1.16) | | -3.01, 1.53 | 0.52 |  |
| *Gender* |  |  | 0.06 | 5.67 |  | |  | 0.15 | 3.78 |
| Female | Ref. |  |  |  |  | |  |  |  |
| Male | **-1.97 (0.83)** | **-3.60, -0.34** | **0.02** |  | -1.38 (0.72) | | -2.8, 0.04 | 0.06 |  |
| Other | -0.02 (1.49) | -2.94, 2.90 | 0.99 |  | 0.13 (1.32) | | -2.45, 2.72 | 0.92 |  |
| *Marital status* |  |  | 0.17 | 7.83 |  | |  | 0.92 | 1.44 |
| Single | Ref. |  |  |  |  | |  |  |  |
| Married or common-law | -1.17 (0.79) | -2.72, 0.38 | 0.14 |  | 0.18 (0.69) | | -1.18, 1.53 | 0.8 |  |
| Divorced | 0.63 (1.54) | -2.38, 3.65 | 0.68 |  | 0.58 (1.33) | | -2.03, 3.18 | 0.67 |  |
| Separated | -1.21 (2.04) | -5.21, 2.79 | 0.55 |  | 0.45 (1.92) | | -3.31, 4.22 | 0.81 |  |
| Widowed | 3.91 (6.2) | -8.24, 16.1 | 0.53 |  | -1.62 (5.47) | | -12.3, 9.1 | 0.77 |  |
| No response | 4.13 (2.24) | -0.26, 8.52 | 0.07 |  | 2.15 (1.98) | | -1.73, 6.03 | 0.28 |  |
| *Education level* |  |  | **0.004** | **15.16** |  | |  | 0.06 | 8.9 |
| < Grade 12 | Ref. |  |  |  |  | |  |  |  |
| High school | -1.22 (1.81) | -4.77, 2.33 | 0.5 |  | -0.06 (1.63) | | -3.26, 3.13 | 0.97 |  |
| College | -1.90 (1.73) | -5.29, 1.49 | 0.28 |  | 0.74 (1.58) | | -2.35, 3.84 | 0.64 |  |
| Undergraduate | -3.06 (1.74) | -6.47, 0.35 | 0.08 |  | -2.02 (1.58) | | -5.12, 1.98 | 0.20 |  |
| Graduate | **-4.78 (1.78)** | **-8.27, -1.29** | **0.008** |  | -2.34 (1.62) | | -5.52, 0.84 | 0.15 |  |
| *Mental health diagnosis* |  |  | 0.59 | 1.05 |  | |  | 0.82 | 0.38 |
| Yes | Ref. |  |  |  |  | |  |  |  |
| No | -0.9 (0.89) | -2.63, 0.84 | 0.312 |  | 0.41 (0.77) | | -1.11, 1.92 | 0.60 |  |
| No response | -0.49 (1.91) | -4.23, 3.25 | 0.797 |  | 0.63 (1.68) | | -2.67, 3.92 | 0.71 |  |
| AIC | 2225.3 | | | | | 2164.4 | | | |
| Residual deviance | 11858 | | | | | 9344.8 | | | |

AIC: Akaike information criterion, *β:* standardized beta coefficient, CI: confidence interval, FCV-19S: Fear of COVID-19 scale, GAD-7: Generalized Anxiety Scale, PHQ-9: Patient Health Questionnaire, Ref.: reference level, SE: standard error.
