# Supplementary material for: Impact of the COVID-19 pandemic on adults accessing specialist psychiatric care: A cross-sectional Canadian analysis
Source: PLoS One. 2026 Apr 15;21(4):e0346913. doi: 10.1371/journal.pone.0346913 (PMC13082661; doi:10.1371/journal.pone.0346913)
Supplement: S2 Table — (DOCX) [file pone.0346913.s002.docx]

**Supplementary Material 2**

**S2 Table.** Bivariate linear regression analyses of Brief-COPE domain subscales on psychological outcomes

| Predictor | Outcome: PHQ-9 | | | Outcome: GAD-7 | | |
| --- | --- | --- | --- | --- | --- | --- |
|  | *β* (SE) | 95% CI | *p*-value | *β* (SE) | 95% CI | *p*-value |
| Brief-COPE: Emotion-focused | 0.11 (0.06) | -0.01, 0.24 | 0.08 | **0.29 (0.05)** | **0.18, 0.39** | **<0.001** |
| F | 3.03 | | | 27.09 | | |
| R^2^ | 0.01 | | | 0.07 | | |
| Brief-COPE: Problem-focused | **-0.14 (0.07)** | **-0.27, -0.01** | **0.04** | 0.03 (0.06) | -0.08, 0.15 | 0.59 |
| F | 4.32 | | | 0.29 | | |
| R^2^ | 0.01 | | | -0.002 | | |
| Brief-COPE: Avoidant | **0.4 (0.08)** | **0.25, 0.55** | **<0.001** | **0.45 (0.07)** | **0.31, 0.58** | **<0.001** |
| F | 26.41 | | | 43.4 | | |
| R^2^ | 0.07 | | | 0.11 | | |

*β:* standardized beta coefficient, Brief-COPE: Brief Coping Orientation to Problems Experienced inventory, CI: confidence interval, GAD-7: Generalized Anxiety Scale, PHQ-9: Patient Health Questionnaire, SE: standard error.
