# Supplementary material for: Impact of the COVID-19 pandemic on adults accessing specialist psychiatric care: A cross-sectional Canadian analysis
Source: PLoS One. 2026 Apr 15;21(4):e0346913. doi: 10.1371/journal.pone.0346913 (PMC13082661; doi:10.1371/journal.pone.0346913)
Supplement: S3 Table — (DOCX) [file pone.0346913.s003.docx]

**Supplementary Material 3**

**S3 Table.** Multiple linear regression of problem-focused coping, COVID-19 fear, and sociodemographic factors on depressive symptoms

| Predictor | Outcome: PHQ-9 | | | |
| --- | --- | --- | --- | --- |
|  | *β* (SE) | 95% CI | *p*-value | χ^2^ |
| Brief-COPE: problem-focused | **-0.15 (0.67)** | **-0.28, -0.02** | **0.025** |  |
| FCV-19S | 0.10 (0.56) | (-0.01, 0.21 | 0.07 |  |
| Age | -0.01 (0.38) | -0.08, 0.07 | 0.9 |  |
| *COVID-19 wave* |  |  | **<.001** | **20.46** |
| Wave 1 | Ref. |  |  |  |
| Wave 2 | 1.41 (-0.95) | 0.45, 3.27 | 0.14 |  |
| Wave 3 | **3.18 (0.89)** | **1.42, 4.93** | **<.001** |  |
| Wave 4 | 1.64 (1.17) | -0.65, 3.94 | 0.16 |  |
| Wave 5+ | -2.01 (1.35) | -4.66, 0.64 | 0.14 |  |
| *Gender* |  |  | 0.06 | 5.71 |
| Female | Ref. |  |  |  |
| Male | -1.99 (0.83) | -3.62, -0.36 | 0.02 |  |
| Non-binary and other | -0.21 (1.49) | -3.14, 2.72 | 0.89 |  |
| *Marital status* |  |  | 0.14 | 8.39 |
| Divorced | Ref. |  |  |  |
| Married or common-law | -2.24 (1.48) | -5.15, 0.66 | 0.13 |  |
| Single | -1.13 (1.56) | -4.18, 1.93 | 1.56 |  |
| Separated | -2.59 (2.33) | -7.19, 2.01 | 0.27 |  |
| Widowed | 3.84 (6.33) | -8.57, 16.3 | 0.54 |  |
| No response | 2.85 (2.67) | -2.39, 8.09 | 0.29 |  |
| *Education level* |  |  | **0.005** | **14.96** |
| < Grade 12 | Ref. |  |  |  |
| High school | -1.11 (1.82) | -4.67, 2.45 | 0.54 |  |
| College | -1.61 (1.74) | -5.02, 1.79 | 0.35 |  |
| Undergraduate | -3.1 (1.74) | -6.50, 0.31 | 0.08 |  |
| Graduate | **-1.61 (1.78)** | **-8.03, -1.05** | **0.01** |  |
| *Mental health diagnosis* |  |  | 0.56 | 1.17 |
| No | Ref. |  |  |  |
| Yes | 0.95 (0.9) | -0.82, 2.71 | 0.29 |  |
| No response | 0.25 (2.02) | -3.71, 4.2 | 0.90 |  |
| AIC | 2186.7 | | | |
| Residual deviance | 11570 | | | |

AIC: Akaike information criterion, *β:* standardized beta coefficient, Brief-COPE: Brief Coping Orientation to Problems Experienced inventory, CI: confidence interval, FCV-19S: Fear of COVID-19 scale, PHQ-9: Patient Health Questionnaire, Ref.: reference level, SE: standard error.
