# Supplementary material for: Impact of the COVID-19 pandemic on adults accessing specialist psychiatric care: A cross-sectional Canadian analysis
Source: PLoS One. 2026 Apr 15;21(4):e0346913. doi: 10.1371/journal.pone.0346913 (PMC13082661; doi:10.1371/journal.pone.0346913)
Supplement: S4 Table — (DOCX) [file pone.0346913.s004.docx]

**Supplementary Material 4**

**S4 Table.** Multiple linear regression of avoidant coping, COVID-19 fear, and sociodemographic factors on depressive symptoms

| Predictor | Outcome: PHQ-9 | | | |
| --- | --- | --- | --- | --- |
|  | *β* (SE) | 95% CI | *p*-value | χ^2^ |
| Brief-COPE: Avoidant | **0.28 (0.08)** | **0.12, 0.44** | **<.001** |  |
| FCV-19S | 0.08 (0.05) | -0.03, 0.18 | 0.17 |  |
| Age | 0.01 (0.04) | -0.06, 0.08 | 0.76 |  |
| *COVID-19 wave* |  |  | **0.001** | **17.78** |
| Wave 1 | Ref. |  |  |  |
| Wave 2 | 1.58 (0.93) | -0.25, 3.4 | 0.09 |  |
| Wave 3 | **2.78 (0.89)** | **1.03, 4.53** | **0.002** |  |
| Wave 4 | 1.87 (1.16) | -0.4, 4.13 | 0.12 |  |
| Wave 5+ | -1.91 (1.32) | -4.49, 0.68 | 0.15 |  |
| *Gender* |  |  | 0.09 | 4.88 |
| Female | Ref. |  |  |  |
| Male | -1.8 (0.83) | -3.43, -0.18 | 0.03 |  |
| Non-binary and other | 0.2 (1.47) | -2.68, 3.07 | 0.89 |  |
| *Marital status* |  |  | 0.56 | 3.93 |
| Divorced | Ref. |  |  |  |
| Married or common-law | -1.27 (1.5) | -4.22, 1.67 | 0.40 |  |
| Single | -0.34 (1.57) | -3.42, 2.74 | 0.83 |  |
| Separated | -1.6 (2.33) | -6.16, 2.97 | 0.49 |  |
| Widowed | 4.29 (6.24) | -7.93, 16.5 | 0.49 |  |
| No response | 2.11 (2.83) | -3.44, 7.67 | 0.46 |  |
| *Education level* |  |  | **0.01** | **13.95** |
| < Grade 12 | Ref. |  |  |  |
| High school | -0.89 (1.79) | -4.39, 2.61 | 0.62 |  |
| College | -1.55 (1.71) | -4.9, 1.8 | 0.37 |  |
| Undergraduate | -2.92 (1.71) | -6.28, 0.44 | 0.09 |  |
| Graduate | **-4.27 (1.76)** | **-7.71, -0.83** | **0.02** |  |
| *Mental health diagnosis* |  |  | 0.6 | 1.02 |
| No | Ref. |  |  |  |
| Yes | 0.88 (0.88) | -0.85, 2.61 | 0.32 |  |
| No response | 0.35 (1.98) | -3.54, 4.24 | 0.86 |  |
| AIC | 2162.2 | | | |
| Residual deviance | 11105 | | | |

AIC: Akaike information criterion, *β:* standardized beta coefficient, Brief-COPE: Brief Coping Orientation to Problems Experienced inventory, CI: confidence interval, FCV-19S: Fear of COVID-19 scale, PHQ-9: Patient Health Questionnaire, Ref.: reference level, SE: standard error.
