# Supplementary material for: Impact of the COVID-19 pandemic on adults accessing specialist psychiatric care: A cross-sectional Canadian analysis
Source: PLoS One. 2026 Apr 15;21(4):e0346913. doi: 10.1371/journal.pone.0346913 (PMC13082661; doi:10.1371/journal.pone.0346913)
Supplement: S5 Table — (DOCX) [file pone.0346913.s005.docx]

**Supplementary Material 5**

**S5 Table.** Multiple linear regression analysis of emotion-focused coping, COVID-19 fear and sociodemographic factors on anxiety symptoms

| Predictor | Outcomes: GAD-7 | | | |
| --- | --- | --- | --- | --- |
|  | *β* (SE) | 95% CI | *p*-value | χ^2^ |
| Brief-COPE: Emotion-focused | **0.24 (0.05)** | **0.13, 0.35** | **<.001** |  |
| FCV-19S | **0.24 (0.05)** | **0.15, 0.33** | **<.001** |  |
| Age | -0.03 (0.03) | -0.1, 0.03 | 0.29 |  |
| *COVID-19 wave* |  |  | 0.1 | 7.79 |
| Wave 1 | Ref. |  |  |  |
| Wave 2 | 0.25 (0.82) | -1.35, 1.86 | 0.76 |  |
| Wave 3 | 1.36 (0.77) | -0.15, 2.86 | 0.08 |  |
| Wave 4 | 1.55 (0.99) | -0.39, 3.49 | 0.12 |  |
| Wave 5+ | -1.23 (1.14) | -3.48, 1.01 | 0.28 |  |
| *Gender* |  |  | 0.17 | 3.59 |
| Female | Ref. |  |  |  |
| Male | -1.33 (0.72) | -2.73, 0.07 | 0.06 |  |
| Non-binary and other | 0.21 (1.28) | -2.3, 2.71 | 0.87 |  |
| *Marital status* |  |  | 0.91 | 1.49 |
| Divorced | Ref. |  |  |  |
| Married or common-law | -0.72 (1.24) | -3.14, 1.7 | 0.56 |  |
| Single | -1.07 (1.3) | -3.61, 1.47 | 0.41 |  |
| Separated | -0.1 (2.19) | -4.59, 3.99 | 0.89 |  |
| Widowed | -4.62 (5.41) | -15.2, 5.99 | 0.39 |  |
| No response | -0.14 (2.41) | -4.86, 4.58 | 0.95 |  |
| *Education level* |  |  | **0.028** | **10.86** |
| < Grade 12 | Ref. |  |  |  |
| High school | 0.22 (1.58) | -2.88, 3.31 | 0.89 |  |
| College | -0.56 (1.53) | -3.56, 2.44 | 0.72 |  |
| Undergraduate | -1.83 (1.54) | -4.84, 1.18 | 0.23 |  |
| Graduate | -2.4 (1.57) | -5.48, 0.68 | 0.13 |  |
| *Mental health diagnosis* |  |  | 0.71 | 0.69 |
| No | Ref. |  |  |  |
| Yes | -0.56 (0.75) | -2.04, 0.91 | 0.45 |  |
| No response | 0.15 (1.72) | -3.22, 3.52 | 0.93 |  |
| AIC | 2086.5 | | | |
| Residual deviance | 8458.5 | | | |

AIC: Akaike information criterion, *β:* standardized beta coefficient, Brief-COPE: Brief Coping Orientation to Problems Experienced inventory, CI: confidence interval, FCV-19S: Fear of COVID-19 scale, GAD-7: Generalized Anxiety Disorder scale, Ref.: reference level, SE: standard error.
