# Supplementary material for: Impact of the COVID-19 pandemic on adults accessing specialist psychiatric care: A cross-sectional Canadian analysis
Source: PLoS One. 2026 Apr 15;21(4):e0346913. doi: 10.1371/journal.pone.0346913 (PMC13082661; doi:10.1371/journal.pone.0346913)
Supplement: S6 Table — (DOCX) [file pone.0346913.s006.docx]

**Supplementary Material 6**

**S6 Table.** Multiple linear regression of avoidant coping, COVID-19 fear and sociodemographic factors on anxiety symptoms

| Predictor | Outcome: GAD-7 | | | |
| --- | --- | --- | --- | --- |
|  | *β* (SE) | 95% CI | *p*-value | χ^2^ |
| Brief-COPE: Avoidant | **0.34 (0.07)** | **0.201, 0.477** | **<.001** |  |
| FCV-19S | **0.21 (0.05)** | **0.12, 0.3** | **<.001** |  |
| Age | -0.02 (0.03) | -0.08, 0.04 | 0.59 |  |
| *COVID-19 wave* |  |  | 0.11 | 7.48 |
| Wave 1 | Ref. |  |  |  |
| Wave 2 | 0.40 (0.82) | -1.2, 1.99 | 0.63 |  |
| Wave 3 | 1.43 (0.77) | -0.09, 2.95 | 0.07 |  |
| Wave 4 | 1.78 (1.0) | -0.18, 3.74 | 0.08 |  |
| Wave 5+ | -0.83 (1.14) | -3.06, 1.4 | 0.47 |  |
| *Gender* |  |  | 0.22 | 3.04 |
| Female | Ref. |  |  |  |
| Male | -1.19 (0.71) | -2.59, 0.21 | 0.1 |  |
| Non-binary and other | 0.4 (1.29) | -2.12, 2.92 | 0.76 |  |
| *Marital status* |  |  | 0.99 | 0.67 |
| Divorced | Ref. |  |  |  |
| Married or common-law | -0.17 (1.29) | -2.69, 2.35 | 0.89 |  |
| Single | -0.65 (1.34) | -3.27, 1.97 | 0.63 |  |
| Separated | -0.65 (2.14) | -4.85, 3.55 | 0.76 |  |
| Widowed | -1.36 (5.44) | -12.0, 9.31 | 0.8 |  |
| No response | -0.12 (2.45) | -4.93, 4.69 | 0.96 |  |
| *Education level* |  |  | 0.08 | 8.24 |
| < Grade 12 | Ref. |  |  |  |
| High school | 0.39 (1.6) | -2.73, 3.52 | 0.81 |  |
| College | -0.18 (1.54) | -3.21, 2.84 | 0.91 |  |
| Undergraduate | -1.6 (1.55) | -4.63, 1.43 | 0.30 |  |
| Graduate | -1.65 (1.59) | -4.76, 1.46 | 0.3 |  |
| *Mental health diagnosis* |  |  | 0.87 | 0.28 |
| No | Ref. |  |  |  |
| Yes | -0.36 (0.76) | -1.85, 1.13 | 0.64 |  |
| No response | 0.12 (1.73 | -3.27, 3.52 | 0.94 |  |
| AIC | 2096.8 | | | |
| Residual deviance | 8590.4 | | | |

AIC: Akaike information criterion, *β:* standardized beta coefficient, Brief-COPE: Brief Coping Orientation to Problems Experienced inventory, CI: confidence interval, FCV-19S: Fear of COVID-19 scale, GAD-7: Generalized Anxiety Disorder scale, Ref.: reference level, SE: standard error.
